# The Impact of Goiter and Thyroid Surgery on Goiter Related Esophageal Dysfunction. A Systematic Review

**DOI:** 10.3389/fendo.2018.00679

**Published:** 2018-11-20

**Authors:** Jesper Roed Sorensen, Steen Joop Bonnema, Christian Godballe, Laszlo Hegedüs

**Affiliations:** ^1^Department of ORL–Head and Neck Surgery and Audiology, Odense University Hospital, Odense, Denmark; ^2^Department of Endocrinology and Metabolism, Odense University Hospital, Odense, Denmark

**Keywords:** goiter, thyroidectomy, esophagus, swallowing, thyroid dysfunction, patient-reported outcomes, systematic review

## Abstract

**Background:** Patients with goiter referred for thyroidectomy report swallowing difficulties. This might be associated with esophageal compression and deviation as this is present in a significant number of patients. Studies on how goiter and subsequently its treatment affect the esophagus are sparse and point in various directions. Our aim was to investigate, through a systematic review, the impact of goiter and thyroidectomy on esophageal anatomy, esophageal physiology, and subjective swallowing dysfunction.

**Methods:** The search period covered 1 January 1975 to 1 July 2018, using the scientific databases PubMed and EMBASE. Inclusion criteria were adult patients with goiter who were either observed or underwent thyroidectomy. Search terms were variations of the terms for goiter, esophagus, swallowing, and dysphagia. From an initial 3,040 titles, 55 full text evaluations led to the final inclusion of 27 papers. Seventeen papers investigated, prospectively, the impact of thyroidectomy on the esophagus, while five observational and five retrospective studies were also included.

**Results:** Esophageal anatomy impairment: Esophageal deviation occurred in 14% and esophageal compression in 8–27% of goiter patients. The prevalence increased with goiter size and with the extent of substernal extension. The smallest cross-sectional area of the esophagus increased by median 34% after thyroidectomy. Esophageal physiology changes: Goiter patients had increased esophageal transit time, positively correlated with goiter size, but unrelated to esophageal motility disturbances. Decrease in the upper esophageal sphincter pressure occurred early after surgery, and normalized within 6 months. Swallowing related patient-reported outcomes: Evaluated by validated questionnaires, swallowing symptoms worsened in the early period after thyroidectomy, but improved after 6 months, as compared to baseline.

**Conclusions:** Thyroidectomy relieved patients with goiter from dysphagia, within 6 months of surgery probably via increase in the cross-sectional area of the esophagus. Attention to the impact by goiter on the esophagus is needed, and balanced and individualized information about the potential benefits and risks of thyroid surgery is crucial in the management of patients with goiter.

## Introduction

Nodularity of the thyroid gland–caused by delicate interactions between genetic and environmental factors such as low iodine intake and cigarette smoking-is common and, depending on definition and type of imaging, affect 20–76% of the population (1–4). The vicinity of the thyroid to vital structures in the neck and upper mediastinum, i.e., the trachea, esophagus, nerves, and blood vessels, combined with a tendency to gradually increase in size, leads to a variety of potential clinical manifestations of the goiter. These include compromised respiration and dysphagia, as well as globus and choking sensation, as some of the most prevalent symptoms (5–9). The positive correlation between goiter size and tracheal compression, and the relief following thyroidectomy is well accepted (10). However, studies on how goiter and subsequently its treatment affect the esophagus are sparse and point in various directions.

Patients with goiter referred for thyroidectomy report swallowing difficulties (11–15) and these may be associated with esophageal compression and deviation, which are abnormalities found to be present in a significant number of patients (8, 16). Thyroidectomy seems to improve both swallowing difficulties (12, 17, 18) and quality of life (19–21) in patients with goiter. The latter improves already 3 months after surgery (19), whereas at least 6 months need to pass before improvements in swallowing symptoms can be expected (11, 22). Comparison of the obviously inhomogeneous studies of the esophagus is almost impossible, which makes it difficult to offer patients with goiter evidence-based information on the surgical effect regarding esophageal compression, dysphagia, and globus sensation.

The aim of the present study was, through a systematic review, to investigate the impact of goiter and thyroidectomy on esophageal anatomy and physiology, and swallowing related patient reported outcomes.

## Methods

### Study design, search terms, and extraction of data

We identified all available English language publications regarding the relationship between goiter and the esophagus, covering the period from 1 January 1975 to 1 July 2018. The search terms used in the scientific databases PubMed and EMBASE were variations of the terms for goiter, esophagus, swallowing, and dysphagia, with MESH terms being Goiter, Esophagus, Deglutition, and Deglutition disorders.

Initially, we screened publication titles and then selected abstracts of relevance. Each identified paper was screened in accordance with the inclusion criteria being: data obtained in humans; age ≥18 years; individuals should undergo thyroidectomy or be offered observation alone; any study should include a minimum of 15 patients. The reference list of each paper was screened for missed publications. The data extracted included number of patients, gender, study design, blinding, inclusion, and exclusion criteria, summary of results, and the presence of major confounders as well as limitations and biases. Biases were assessed for the individual studies using the “Cochrane Risk of Bias tool” (23).

## Results

### Study selection and characteristics

**Figure 1:**
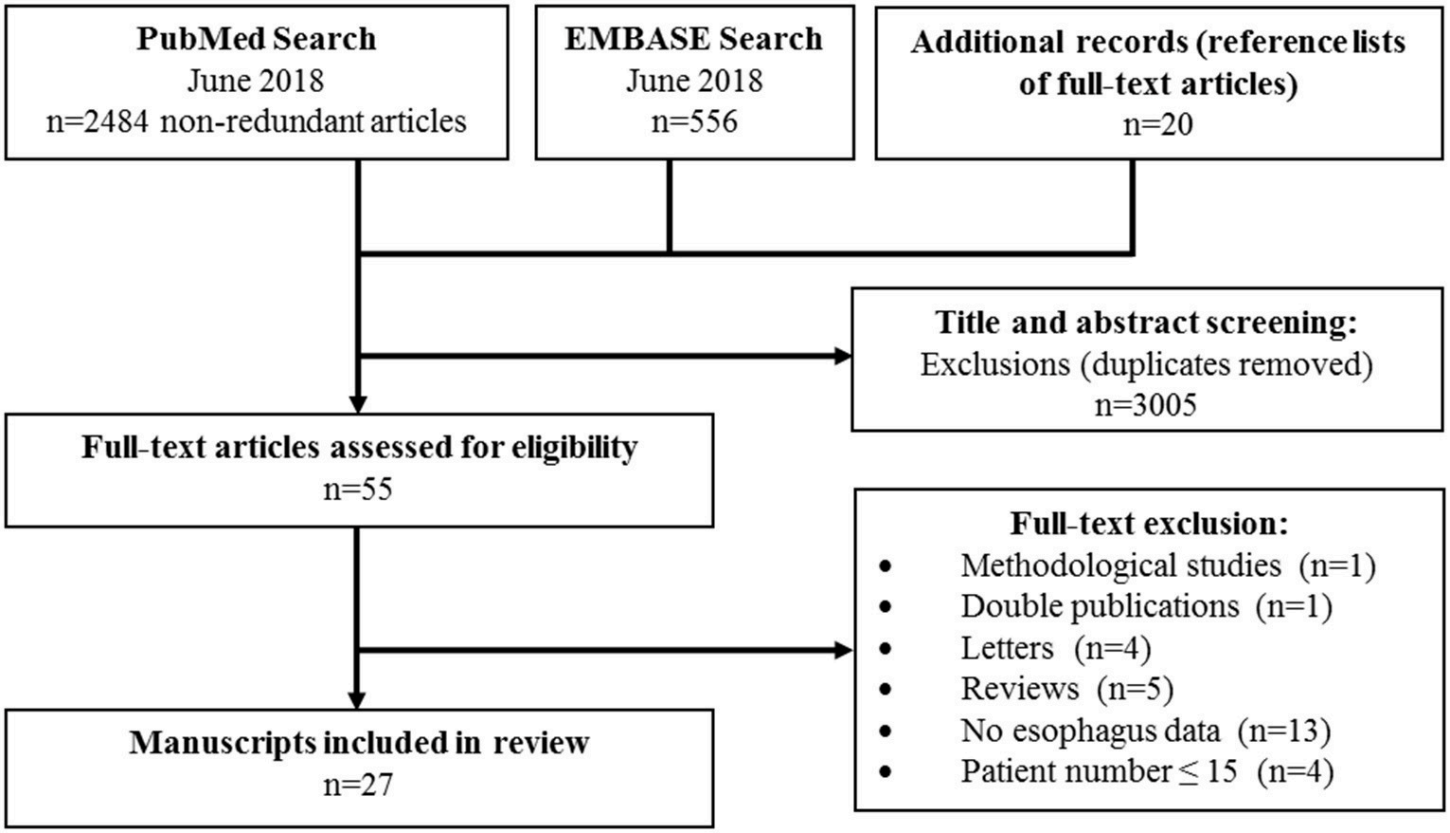
Flow diagram of manuscript selection of final inclusion and final inclusion of full-text manuscripts.

From PubMed and EMBASE 3,040 relevant titles were included, supplemented by 20 articles obtained from the reference lists of included papers (Figure 1). We excluded 3,005 papers after review of titles and abstracts, leaving 55 for full text evaluation. Twenty-eight full text papers failed to meet the inclusion criteria, resulting in 27 papers for the final analyses.

Applying the “Cochrane Risk of Bias tool” across studies (23), we found a considerable risk of bias as many studies failed to describe inclusion procedures, include control groups, randomize for treatment, or blind observers or participants (Table 1). As a consequence, we made no attempt to perform a meta-analysis. Most of the included studies (Tables 2, 3) were small, with 60 or fewer individuals in 14 of the 27 studies. Only one study applied randomization (15) and three studies used some blinding of participants, personnel or the evaluated outcomes (25, 30, 31). There were five observational studies, 17 studies investigating the impact of thyroidectomy, and five retrospective studies with two studies investigating thyroidectomy (Tables 2, 3). As evident from the heterogenous studies, the level of evidense range from low to moderate (Table 4).

**Table 1:** Risk of bias summary in the 27 studies included for review.

**References**	**Random sequence generation**	**Allocation concealment**	**Blinding of participants and personnel**	**Blinding of outcome assessment**	**Incomplete data**	**Selective reporting**	**Other bias**
Shin et al. (16)	?	?	?	?	?	?	?
Netterville et al. (8)	?	?	?	?	?	?	?
Mackle et al. (24)	?	+	?	?	?	?	?
Brinch et al. (25)	?	?	+	+	?	?	+
Nam et al. (26)	–	+	?	?	+	+	?
Jorgensen et al. (27)	?	?	?	?	?	?	?
Glinoer et al. (28)	?	?	?	?	?	?	?
Scerrino et al. (13)	?	?	?	?	?	?	?
Scerrino et al. (29)	?	?	?	?	–	?	?
Sorensen et al. (30)	?	?	+	+	?	?	?
Arakawa-Sugueno et al. (31)	–	–	–	+	?	?	?
Gohrbandt et al. (32)	–	–	–	–	+	?	?
Fiorentino et al. (33)	?	?	?	?	–	?	?
Hyun et al. (34)	?	?	?	?	+	+	–
Rodrigues et al. (35)	–	–	–	–	?	?	?
Sabaretnam et al. (12)	?	?	?	?	?	–	–
Greenblatt et al. (17)	?	?	?	?	?	?	?
Maung et al. (18)	?	?	?	?	?	?	?
Lombardi et al. (11)	?	?	?	?	–	?	?
Holler and Anderson (36)	?	?	?	?	+	+	+
Tae et al. (22)	?	–	?	?	?	?	?
Lombardi et al. (37)	?	?	?	?	+	?	?
Lombardi et al. (15)	?	?	?	?	?	?	?
Grover et al. (38)	?	?	?	?	?	?	?
Burns and Timon (39)	+	?	?	?	+	?	?
Krekeler et al. (40)	?	?	?	?	+	+	?
Pereira et al. (41)	+	+	–	–	?	?	?

*?, Unclear risk of bias; +, low risk of bias; −, high risk of bias*.

**Table 2:** Studies investigating the effect of goiter on the esophagus anatomy abnormalities and the esophageal physiology changes.

**References**	**Design**	***n***	**Cohort**	**Methods**	**Results**
Shin et al. (16)	RE	198	Age: 59 (22–89) years. Goiter: 143 g	CXR, CT	Esophageal compression/deviation: 8%/14% of patients with goiter
Netterville et al. (8)	RE	23	Age: 59 (32–91) years. Goiter: 148 (38–426) g	CXR, CT, MRI	Esophageal compression or deviation: 27% of patients with substernal goiter
Mackle et al. (24)	OB	26	Age: mean 57 years. Goiter: NA	CT	Esophageal deviation: 95% of patients with substernal goiter
Brinch et al. (25)	TH	64	Age: 52 (21–77) years. Goiter: 57 (14–642) mL	MRI, ThyPRO	SCAE increased from 95 mm^2^ (47–147) to 137 (72–286) mm^2^ in combination with decrease in goiter symptoms from 40 to 10 points at 6 months after surgery
Nam et al. (26)	OB	175	Age: 54 ± 12 years. Goiter: Single nodule	US	Globus sensation: 48 %. Nodules >3 cm and nodules located horizontally and anteriorly to the trachea are associated with globus sensation
Jorgensen et al. (27)	OB	74	Age: 40 (20–74) years. Goiter: NA	Scintigraphy	Increased MTT for patients with large goiters compared to healthy controls, and patients with small goiters, or enlarged atrium
Glinoer et al. (28)	OB	148	Age: 42 ± 13 years. Goiter: 33 ± 22 g	Scintigraphy	39% of patients with goiter had abnormal MTT MTT prolonging correlates with substernal goiter
Scerrino et al. (13)	TH	36	Age: 49 (26–65) years. Goiter: < 60 mL	Manometry, MSIS	Swallowing impairment: Baseline: 78% of patients, after 1 month: 64% of patients. Decrease in UES 1 month after total thyroidectomy
Scerrino et al. (29)	TH	36	Age: 48 ± 6 years. Goiter: < 60 mL	Manometry, MSIS	Swallowing impairment: 42 % of patients 18–24 months after surgery. UES approaches preoperative values 18–24 months after surgery
Sorensen et al. (30)	TH	33	Age: 60 ± 12 years. Goiter: 50 (8–607) g	Manometry, ThyPRO	UES increases after surgery 6 months after surgery in combination with decrease in goiter symptoms from 39 (2–61) to 5 (0–52) points at 6 months after surgery
Arakawa-Sugueno et al. (31)	TH	54	Age: NA Goiter: NA	Vide-endoscopic evaluation	Seven days after thyroidectomy, dysphagia in 87 % of patients with abnormal laryngeal mobility (ALM) and 44% of patients with normal laryngeal mobility (NLM). 60 days after surgery dysphagia in 67% of ALM patients and 25% of NLM patients
Gohrbandt et al. (32)	TH	53	Age: 52 ± 13 years. Goiter: NA	US	Reduced laryngeal mobility for at least 6 months after surgery for men, while women have fully recovered at 6 months after surgery
Fiorentino et al. (33)	TH	34	Age: 51 (21–77) years. Goiter: 58 ± 18 mL	VFSS	No change in hyoid elevation, epiglottic tilting or stasis of food bolus after thyroidectomy
Hyun et al. (34)	TH	47	Age: NA Goiter: NA	Swallowing movement, SIS	Transaxillary endoscopic thyroidectomy less SIS score, lower muscle adhesion, and greater hyoid bone movement than following regular thyroidectomy
Rodrigues et al. (35)	RE	113	Age: NA Goiter: >40 mL	Chart-review	Reflux laryngitis; cervical goiter: 21% vs. substernal goiter: 42% Digestive-compressive symptoms; cervical goiter: 77% vs. substernal goiter: 85%

*RE, Retrospective; OB, Observational; TH, Thyroidectomy; NA, Not available; CXR, Chest X-ray; ThyPRO, Thyroid-specific Patient-reported Outcome; SCAE, Smallest cross-sectional area of the esophagus; US, Ultrasound; MTT, Mean esophageal transit time; MSIS, Modified Swallowing Impairment Score; UES, Upper esophageal sphincter pressure; VFSS, Videoflurographic swallowing study; SIS, Swallowing Impairment Score*.

**Table 3:** Studies investigating the effect of goiter on the swallowing-related patient reported outcomes.

**References**	**Design**	***n***	**Cohort**	**Methods**	**Results**
Sabaretnam et al. (12)	TH	224	Age: mean 38 years. Goiter: mean 85 g	SWAL-QOL	SWAL-QOL: 8 of 11 domains affected before surgery all of which improved 6 months after surgery.
Greenblatt et al. (17)	TH	116	Age: 49 ± 13 years. Goiter: 36 ± 34 g	SWAL-QOL	SWAL-QOL: 9 of 11 domains affected before surgery with improvement in 8 of the 9 domains after surgery. No difference between hemi- and total thyroidectomy.
Maung et al. (18)	TH	41	Age: mean 48 years. Goiter: ≥WHO gr. II	GETS	GETS: 3 months after surgery: 2 of 12 items improved 12 months after surgery: 6 of 12 items improved
Lombardi et al. (11)	TH	110	Age: 47 ± 13 years. Goiter: 33 ± 22 g	SIS	SIS ≥1 before: 47% of patients. After 1 week: 74% of patients. After 1 month: 64% of patients. After 3 months: 48% of patients. After 1 year: 20% of patients.
Holler and Anderson (36)	OB	59	Age: 19–73 years. Goiter: NA	MSIS	MSIS: Swallowing complaints: 43% (at least some of the time) and 27% (often or always).
Tae et al. (22)	TH	111	Age: mean 48 years. Goiter: nodule < 5 cm	SIS	Swallowing symptom score: Increase 1 day, 1 week, 1 month, and 3 months after surgery. At 6 months after surgery values returned to presurgical values. No difference between robotic- and conventional thyroidectomy
Lombardi et al. (37)	TH	127	Age: 43 ± 11 years. Goiter: 26 ± 9 g	SIS	Mean SIS. Baseline: 0.5 points. After 1 week: deterioration to 10.3 points. After 1 month: 6.0 points. After 3 months: 2.8 points
Lombardi et al. (15)	TH	53	Age: NA Goiter: < 30 mL	SIS	VAT had a significantly lower and improved SIS score at 1 week after surgery compared to conventional thyroidectomy. No change at 1 or 3 months after surgery
Grover et al. (38)	RE	202	Age: 55 ± 16 years. Goiter: mean 78 g	SIS	One year after total thyroidectomy or completion thyroidectomy: 41% have normal SIS score (< 10 points), 28% have moderately affected score (28%) and 31% have severely affected score (>16 points). Scores did not change beyond 1 year.
Burns and Timon (39)	TH	58	Age: mean 40 years. Goiter: 62 g	VAS (0–10 points)	Globus sensation: Baseline 58% (mean 5.2 points), significant improvement 3–6 months after surgery 6% (mean 1.1 point)
Pereira et al. (40)	TH	26	Age: 46 ± 4 years. Goiter: 2.2 ± 1.4 cm	Interview	80% had at least 1 swallowing-related symptom 2 weeks after surgery, 42% at 6 weeks, and 17% at 6 months
Kahrilas et al. (41)	RE	120	Age: 58 ± 3 years. Goiter: NA	Chart review	Dysphagia in 2 of 60 patients with goiter before thyroidectomy and in 9 of these patients 4 years after surgery.

*RE, Retrospective; OB, Observational; TH, Thyroidectomy; SWAL-QOL, Swallowing quality of life questionnaire; GETS, Glascow-Edinburgh Throat Scale; NA, Not available; VAT, Video-Assisted Thyroidectomy; SIS, Swallowing Impairment Score; VAS, Visual Analog Score; MSIS, Modified Swallowing Impairment Score*.

**Table 4:** Recommendations based on strength of evidence (50).

**Theme**	**Physiological effect**	**Level of evidence**	**Sources (references)**
Esophageal anatomy	Patients with goiter can have esophageal deviation and/or compression. The prevalence increases if the goiters increase in size or becomes substernal	Moderate (several studies with some limitations)	(8, 16, 24, 25)
	After thyroidectomy some esophageal deviation might persist as normal physiology, but esophageal compression is relieved	Moderate (several studies with some limitations)
Esophageal physiology	Patients with goiter may show increased esophageal transit time, correlating with the goiter size. This seems not related to esophageal motility disturbances	Low (one or more studies with severe limitations)	(13, 27–30)
	Some esophageal motility disturbance may persist in the weeks after surgery, but not for longer than 6 months after surgery	Low (one or more studies with severe limitations)
Patient reported outcome	Patients with goiter have a high prevalence of swallowing symptoms.	Moderate (several studies with some limitations)	(11–13, 15, 17, 18, 22, 29, 34, 36–38)
	Swallowing symptoms might deteriorate in the weeks and months after thyroidectomy, but from 6 months swallowing symptom are reduced to a lower level than preoperatively. A subgroup of patients might have persistent swallowing complaints	Low (one or more studies with severe limitations)

*Moderate level of evidence: Further research is likely to have an important impact on our confidence in the estimate of effect and may change the estimate. Low level of evidence: Further research is very likely to have an important impact on our confidence in the estimate of effect and is likely to change the estimate*.

### Esophageal anatomy abnormalities

Affection of the esophagus was a common finding among patients with goiter (Table 2). Four studies evaluated, by chest X-ray, CT, or MRI, the prevalence of esophageal compression and/or deviation in patients undergoing thyroidectomy (8, 16, 24, 25). A study of 198 patients found esophageal deviation in 14% of individuals, and esophageal compression in even fewer (8%) (16). A higher prevalence of esophageal compression was found in another study of only 23 patients, with six patients (26%) having esophageal compression (8). A third study found 24 of 26 patients (95%) with substernal goiter to have esophageal midline deviation (24). All the above studies had shortcomings, such as unclear definitions of either esophageal deviation and/or compression.

A more recent study (25), with well-defined definitions of both esophageal deviation and compression, evaluated 65 well-characterized goiter patients undergoing neck MRI before and 6 months after thyroidectomy. In that study, the median esophageal deviation decreased significantly from 4 mm (range: 0–23 mm) to 3 mm (range: 0–10 mm). More importantly, the median smallest cross-sectional area of the esophagus (SCAE) increased from 95 mm^2^ (range: 47–147 mm^2^) at baseline to 137 mm^2^ (range: 72–286 mm^2^) after surgery, corresponding to a median increase of 34% (range, −17 to 253%) in SCAE (25). The anterior-posterior depth of the esophagus did not change significantly following surgery, while the median-lateral width of the esophagus achieved a small but significant increase from a median of 15 mm (range: 10–21 mm) to 17 mm (range: 12–24) after surgery (Table 2). The esophagus had a small midline deviation, and this persisted after surgery. More importantly, there was an increase in the cross-sectional area of the esophagus following surgery, and a change from a more round to a more ellipsoid shape. The preoperative volumes of the thyroid and the SCAEs were significantly inversely correlated, with reduction in SCAE of 0.35 mm^2^ for every 10% increase in goiter volume. In addition, the SCAE increased by 1% for every 10% increase in the weight of the removed thyroid tissue (25). This implies that patients with the initially largest goiters also experienced the most pronounced improvements in SCAE, following thyroid surgery.

In relation to the impact of individual nodules on esophageal anatomy, one study found globus sensation to be present in 48% of patients. Nodules >3 cm in diameter, and/or located anteriorly and horizontally to the trachea were more often present in patients with globus sensation than in patients with smaller nodules or nodules located laterally to the trachea (26).

### Esophageal physiology changes

The swallowing function had been investigated by various objective methods (Table 2). Using scintigraphic methods for assessment, an increase in the esophageal transit time by 2–7 s were demonstrated in patients with a large goiter (>7 cm long or >3 cm in depth, evaluated by ultrasound), as compared to smaller goiters (27). Another study, also using nuclear medicine techniques, found 39% of goiter patients to have increased esophageal transit time (28).

A study of 36 patients identified a decrease in the upper esophageal sphincter (UES) pressure, using water manometry, in the early period after total thyroidectomy (13). However, these findings were normalized 18–24 months after surgery in a subsequent study of the same patient cohort (29). Another study of 33 patients, found normal esophageal motility, both before and 6 month after surgery (30). Only the UES basal pressure increased significantly from 71 to 88 mmHg 6 month after surgery, the latter value being within the normal limits (30). In our study, using the Chicago classification version 3.0 for assessing motility disturbances (42), only one of the 33 patients with a benign goiter (3%) had a minor peristaltic disorder preoperatively, defined as >50% weak or failed coordinated esophageal muscle contractions during 10 swallowing events (30). Two patients (6%) fulfilled these criteria 6 months after surgery. Importantly, as patient characteristics as well as the manometric examination techniques (i.e., water manometry and high resolution esophageal manometry), differed between the studies, a direct, comparison across studies is not meaningful. Nevertheless, it seems that decrease in the UES pressure potentially may be present in the early period after surgery as the goiter is removed. This issue is of no greater concern beyond 6 months after surgery.

A few studies have investigated the hyoid bone movement, epiglottic tilting, and laryngeal mobility in goiter patients (31–35). A study of 54 patients demonstrated a preoperative association between abnormal laryngeal mobility and the prevalence of dysphagia in patients with goiter (31). In the immediate weeks after surgery the laryngeal mobility was reduced, with recovery at 6 months postoperatively (32). Another study found the hyoid bone movement to be unchanged after surgery in an investigation of 34 patients (33). In a study with 54 patients divided into two groups using neither randomization nor blinding, trans-axillary thyroidectomy was associated with a lower degree of neck muscle adhesion and a greater hyoid bone movement, as compared with conventional thyroidectomy (34). The prevalence of reflux laryngitis was also found lower in patients with cervical compared to substernal goiter, at 21 vs. 42%, respectively (35).

### Swallowing related patient-reported outcomes

Three studies, using validated swallowing specific questionnaires, have investigated the patients' perception of swallowing before and after thyroidectomy (Table 3) (12, 17, 18). By using the “swallowing related quality of life questionnaire,” eight (12) and nine (17) of 11 swallowing related domains were affected in studies of 116 and 224 patients with goiter, respectively, all of whom were referred for thyroidectomy. Using the validated Glasgow-Edinburgh throat scale, only two of 12 items improved at 3 months after surgery, while six items had improved after 12 months (18).

Nine studies, the largest including more than 100 patients, used the non-validated swallowing impairment score or modified versions hereof (11, 13, 15, 22, 29, 34, 36–38). Prior to thyroidectomy, the prevalence of dysphagia varied between 48 and 78% (11, 13, 36). At 1 week, 1 month, and 3 months after thyroidectomy, three studies found increased prevalence (i.e., worsening) of swallowing symptoms compared to the preoperative level (11, 22, 37), while a single study of 53 patients found a decreased level of dysphagia (15). However, at 6 months or later after surgery, patients had fewer symptoms than at baseline (11, 22, 29), although a subgroup had persistent swallowing complaints (38). Studies using other examination techniques, such as qualitative analyses, supported these findings (39–41). The impact on swallowing symptoms seemed uninfluenced by the surgical technique or whether hemi- or total thyroidectomy was performed (15, 17, 18, 22).

Two recent studies, carried out by our group on the same patient cohort, assessed the impact of thyroidectomy and esophageal dysfunction by use of the well-validated disease-specific ThyPRO questionnaire (25, 30). Goiter patients had a significantly affected mean Goiter Symptom score of 40 points at baseline (maximum of 100 points equals severe symptoms), which improved significantly to a mean of 10 points postoperatively (25) (minimum of 0 points equals no symptoms) (Table 2). All 11 items of the Goiter Symptom scale improved 6 months after surgery. The items related to swallowing difficulties, such as “discomfort swallowing,” underwent a significant score reduction from a mean of 1.8 points (maximum of 4 points) to 0.3 points, postoperatively. Similarly, the mean score for “difficulty swallowing” decreased significantly from 1.2 to 0.2 points after surgery, while “Globus sensation” decreased from 2.3 to 0.7 points.

## Discussion

Studies using objective measures of the effect of goiter on the esophagus are limited. Nevertheless, the existing literature suggests that presence of goiter is associated with both esophageal deviation and compression, and that this is relieved by surgery (8, 16, 24, 25). In addition, goiter patients may show a prolonged passage and some impact on the esophageal peristaltic movement and/or sphincter pressure, as determined by either nuclear medicine techniques or various forms of esophageal manometry (13, 27, 28, 30). Importantly, surgery does not cause deterioration in the above mentioned parameters.

Objective analyses of the esophagus support the high prevalence of preoperative swallowing difficulties as reported by 48–78% of the patients (11, 13, 36). Evaluated by patient reported outcomes, reports on swallowing symptoms during the first 3 months after surgery have shown conflicting results (11, 13, 15, 22, 37). In fact, some studies have shown deterioration until 6 months after surgery (11, 22, 37). More consistently, all studies demonstrate significant improvement of the swallowing symptoms beyond 6 months after surgery (11, 12, 17, 22, 38), although a subgroup of patients have persistent complaints more than 1 year after surgery (38). More generally, the Goiter Symptom Score, extracted from the ThyPRO instrument, improved after surgery, with significantly reduced globus sensation and swallowing difficulties (25, 30). However, symptom assessment obtained by questionnaires cannot be performed with blinding of the patients, which may have influenced the outcome in all studies of this kind.

Further studies are needed to examine the relation between goiter and the esophagus, and the effect of treatment. In particular, it is of interest to compare surgical and non-surgical techniques–such as radioiodine therapy (43) and ultrasound-guided thermal procedures in the form of e.g., laser or radiofrequency ablation (44). These non-surgical ablation techniques are less prone to side-effects such as voice changes and postoperative hypothyroidism (45–48), while the potential effects on the esophagus remain unknown. Awaiting such studies, and comparison with the long-established surgical approaches, leaves a gap in our ability to provide individual-tailored therapy for goiter patients.

## Study limitations

Although inclusion of solely English language publications theoretically might have caused bias, this does not seem to be the case (49). Women constitute the majority of patients, de facto and in the cited literature. This means that some of the conclusions may not be as valid for male as for female goiter patients. The majority of the identified studies contained a limited number of patients, and many of the intervention studies are hampered by loss to follow-up in 20% of patients (15, 17, 33), or higher (11, 37, 38), especially in studies of esophagus motility due to patient discomfort associated with these procedures (9).

Comparing studies of mixed patient populations regarding age, setting of recruitment, diagnosis i.e., thyroid malignancy, hyper- and hypothyroidism, and benign nodular goiter, complicates interpretation of the results. The fact that patients were included from highly selected populations and from both primary, secondary and tertiary centers, as well as from surgical and medical departments constitute additional limitations. Needless to say, it is unknown to which degree these influence our conclusions and generalizability of the manuscript.

### Implications for the future

Future studies should compare ^131^I therapy and ultrasound-guided thermal ablation techniques with various surgical techniques for goiter ablation, as for the effect on the esophagus. Such studies should be performed using validated swallowing and quality-of-life-related questionnaires, with measurements of the esophageal anatomy and function as the potential primary endpoints. Follow-up should be a minimum of 6 months, since improvement of swallowing dysfunction at an earlier time point cannot be expected, at least not after surgical goiter ablation.

## Conclusions

Patients with goiter benefit substantially from thyroidectomy, with profound improvements in dysphagia and increase in the cross-sectional area of the esophagus. However, in most patients the improvements do not occur before 6 months after thyroidectomy. In fact, deterioration in dysphagia may be seen in the early period after surgery. Studies evaluating the long-term status of these parameters, and comparing surgical and non-surgical goiter treatment techniques, are warranted.

## Author contributions

All authors (JS, CG, SB, and LH) contributed in the conception of design, interpretation, revising the manuscript critically, and approval of the final manuscript. JS contributed with acquisition and analysis of data, and drafting the work.

### Conflict of interest statement

The authors declare that the research was conducted in the absence of any commercial or financial relationships that could be construed as a potential conflict of interest.
